# Demography_Lab, an educational application to evaluate population growth: Unstructured and matrix models

**DOI:** 10.1002/ece3.7170

**Published:** 2021-01-19

**Authors:** Julio Arrontes

**Affiliations:** ^1^ Depto. Biología de Organismos y Sistemas University of Oviedo Oviedo Spain

**Keywords:** demographic stochasticity, environmental stochasticity, matrix model, population model, sampling error, sensitivity analysis, stochastic model

## Abstract

Training in Population Ecology asks for scalable applications capable of embarking students on a trip from basic concepts to the projection of populations under the various effects of density dependence and stochasticity. Demography_Lab is an educational tool for teaching Population Ecology aspiring to cover such a wide range of objectives. The application uses stochastic models to evaluate the future of populations. Demography_Lab may accommodate a wide range of life cycles and can construct models for populations with and without an age or stage structure. Difference equations are used for unstructured populations and matrix models for structured populations. Both types of models operate in discrete time. Models can be very simple, constructed with very limited demographic information or parameter‐rich, with a complex density‐dependence structure and detailed effects of the different sources of stochasticity. Demography_Lab allows for deterministic projections, asymptotic analysis, the extraction of confidence intervals for demographic parameters, and stochastic projections. Stochastic population growth is evaluated using up to three sources of stochasticity: environmental and demographic stochasticity and sampling error in obtaining the projection matrix. The user has full control on the effect of stochasticity on vital rates. The effect of the three sources of stochasticity may be evaluated independently for each vital rate. The user has also full control on density dependence. It may be included as a ceiling population size controlling the number of individuals in the population or it may be evaluated independently for each vital rate. Sensitivity analysis can be done for the asymptotic population growth rate or for the probability of extinction. Elasticity of the probability of extinction may be evaluated in response to changes in vital rates, and in response to changes in the intensity of density dependence and environmental stochasticity.

## Graphical abstract

Demography_Lab is an educational application to evaluate the growth of populations. The application uses stochastic unstructured and matrix models and allows for the independent implementation of environmental and demographic stochasticity and sampling error. A large variety of life cycles may be accommodated.

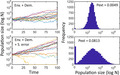

## 1 INTRODUCTION

Students get lost very often with demographic models, particularly with parameter‐rich models or with those with a nontrivial mathematical skeleton. Teaching Population Ecology has to cope with an apparent atavic fear of mathematical expressions in Biology students. Applied Population Ecology demands considerable knowledge and skills in population modeling (see, e.g., Botsford et al., 2019). However, learning of simple models, with a limited utility for management of real populations, is a useful way to introduce mathematical concepts and the basic procedures to evaluate the future of populations. In simple models, the population state is defined by a single variable (e.g., population size), individuals are all considered identical, and demographic rates are averaged across individuals. They are unstructured models. Training in basic concepts includes aspects which are rarely relevant in conservation. This may be the case for overcompensation in density dependence (e.g., Ripa & Heino, 1999). The analysis of replacement curves under contest or scramble competition, or the analysis of the conditions for the existence of cycles or even chaos in deterministic models, may all have a theoretical interest, but limited practical relevance in managing populations. It is clear that most of extinction of contemporary concern occurs from small population size after long periods of decreasing population abundance (Collen et al., 2011). The analysis of simple deterministic and/or unstructured models, however, has an interest in explaining general principles (Hastings, 2005). The consideration of simple models as strategic, explanatory, models dates back at least to Holling (1966) and May (1973). Although not used in the same way as null hypothesis in inferential statistics, simple models may be used by the students as a reference against which the behavior of real populations, or more realistic models, may be compared. Burch (2018) includes the use of simple models in his ten principles for teaching Demography, particularly in introductory courses. Evans et al. (2013), however, suggest that simple models might be an obstacle for the progress of Ecology. As concluded by Evans et al. (2013; but see also Botsford et al., 2019), to understand and predict the behavior of complex ecological systems, models need to incorporate all relevant processes. Structured population models are more realistic and include an age, size, or stage structure (see basic concepts in Caswell et al., 1997). Individuals at each class may have specific demographic rates, which might be affected differently by the environment.

There are some well‐known, excellent, and respected programs to evaluate the future of populations. Just to mention some of the most widespread used and cited, Vortex (Lacy & Pollak, 2020), Ramas (Akçakaya et al., 1999) or popBio (Stubben & Milligan, 2007). Another alternative for teaching demography may be the use of general purpose software for modeling, particularly R and MATLAB. A large number of functions are available for demographic analysis in both environments (e.g., Bernstein, 2003). However, the learning curve is steeper as this software is not particularly friendly for the beginner. And it is important to separate learning of demographic concepts from the learning of programs, applications or code. Demography_Lab is an educational application aimed at graduate and postgraduate students which was designed to serve as a help in teaching Population Ecology. Demography_Lab is not intended to be an alternative to those programs. It allows for step learning, scaling up from the most basic, albeit unrealistic, models to very complex models. Demography_Lab is written in MATLAB code (The MathWorks Inc.). The application is a training tool with a double function, learning of concepts of demography of wild populations and to serve as an introduction to the management of species with an interest in conservation. Demography_Lab has been routinely used in introductory and advanced courses of Ecology at the University of Oviedo (Spain) for the last 8 years. Demography_Lab covers a gap because while ending in full immersion in the analysis of complex models, it permits step‐by‐step learning for the beginner; and no previous knowledge of programing languages is required. Additional interest of Demography_Lab relies in that it is free; allows for complete control of all conditions in the simulations, particularly the sources of stochasticity; and is more user‐friendly than working directly with R or MATLAB.

Three sources of variability are influencing the output of population models: environmental stochasticity, demographic stochasticity, and sampling error in obtaining demographic data (see a summary in Lande et al., 2003). Widely defined, environmental stochasticity refers to temporal variation in extrinsic factors to the populations with some effect in vital rates (Legendre, 2020). Factors may be of very diverse nature, such as density of predators, weather, or food availability. In general, increased environmental variance has a negative effect on population growth (e.g., Legendre, 2020), in part due to a mere Jensen's inequality effect due to geometric averaging of population growth (Denny, 2017; Ruel & Ayres, 1999). The exact influence of environmental stochasticity might only be correctly evaluated if correlation among vital rates in the projection matrix is considered (Doak et al., 2005). The possibility of including temporal correlation of environments also merits attention, as it may have dramatic effects on population growth (Johst & Wissel, 1997; Ripa & Lundberg, 1996; Schwager et al., 2006). Demographic variance has been defined as expected variance in individual fitness within a projection interval (Lande et al., 2003). Demographic variance is a consequence of the random nature of the processes affecting individuals during the projection interval or a consequence of nonpermanent and random differences among individuals (e.g., May, 1973; Shaffer, 1981; Kendall & Fox, 2003; see Legendre, 2020, for a recent description and definitions). Sampling error refers to measurement error or uncertainty in vital rates due limited sample sizes, or nonrepresentative samples, used to extract demographic information from populations. Sampling‐related errors and bias may be increased in populations with a conservation concern as very often these populations are rare and difficult to find (e.g., Thompson, 2004). Availability of data influences uncertainty in the estimation of parameters in stochastic demographic models (Doak et al., 2005). Including observation errors in models considerably influences intervals for parameter estimates (e.g., Newman & Lindley, 2006). Seasonal or periodic environments assume predictable variation in vital rates with time and may be included in both deterministic and stochastic simulations (Caswell, 2001).

Models in Demography_Lab are always stochastic, but deterministic simulations are possible to serve as background dynamics. The user always retains a direct and full control on all demographic and environmental variables. Inputs are a set of demographic parameters and environmental conditions. Demography_Lab operates by evaluating the changes in population size with time after projecting the population for a number of time steps. The application calculates a very large number of trajectories and from these, it extracts the probability of reaching user‐given density thresholds. Demography_Lab uses difference equations or projection matrices operating in discrete time. The models are solved numerically by iteration for a fixed number of time steps. Demography_Lab has two modules. Both allow for the evaluation of future growth and the probability of extinction of populations. The elementary module, Unstructured, is aimed at basic learning, working with populations lacking an age or stage structure. Basic effects of environmental and demographic stochasticity may be evaluated with the Unstructured module. The advanced module, Structured, may be used to evaluate populations with different classes of individuals. More advanced options and the effect of sampling error are evaluated in the Structured module. The student may do a complete trip, with major gains which may be summarized as: (a) the understanding of population parameters and the practical implications of population size, and their ranges of variation, on the future of the populations; and (b) the understanding of how variability in vital rates and the role of different sources of stochasticity influence the viability of the populations.

Sensitivity analysis is a crucial part of the construction of a model (Caswell, 2001, 2019). It may serve to evaluate alternative management strategies, to prepare more efficient sampling designs or to test the model itself. No explicit sensitivity analysis is included in the Unstructured module, though still sensitivity of the probability of extinction to changes in the conditions of the simulation may be evaluated. A variety of techniques is included in the Structured module, from the standard sensitivity and elasticity of asymptotic population growth rate, to the sensitivity of the probability of the extinction to changes in the level of stochasticity or intensity in density dependence. Sensitivity analysis may be done using realistic ranges of variation for almost any demographic parameter, for example, assuming different potential ranges for every vital rate or matrix entry.

Inspiration to build the application came from Caswell (2001) and Lande et al. (2003). Pieces of MATLAB code to extract population parameters and to estimate confidence intervals, the algorithms for the bootstrap, and the steps to construct the projection matrix were all extracted from Caswell's book (2001).

## 2 UNSTRUCTURED MODELS

The application evaluates the growth of populations by using simple unstructured discrete‐time models. The module is a tool to sequentially introduce the student to variability in population growth rates, stochasticity, and the nature of the environment. Four different models are used: exponential, Ricker, Beverton–Holt, and ceiling. Detailed descriptions and major properties of these models may be found in almost any textbook in Population Ecology (e.g., Turchin, 2003). The exponential mode assumes unbounded population growth, with no density‐dependent regulation. The other models assume the existence of density dependence and a carrying capacity.

(1) $$\text{Exponential}:\;N_{t+1}=RN_{t}$$

(2) $$\text{Ricker}:\;N_{t+1}=N_{t}R^{\left(1‐N_{t}/K\right)}\equiv N_{t}e^{r(1‐N_{t}/K)}$$

(3) $$\text{Beverton}‐\text{Holt}:\;N_{t+1}=\frac{RN_{t}}{1+\frac{R‐1}{K}N_{t}}$$

(4) $$\text{Ceiling}:\;N_{t+1}=R\min\left(N_{t},K\right)$$

In all cases, *R* is the multiplicative population growth rate, *K* is carrying capacity, *r* = ln*R*, and *N_t_* and *N_t_*_+1_ are population sizes at times *t* and *t* + 1 (i.e., before and after the projection interval). In the ceiling model, density dependence limits the number of reproductive individuals, not the number of recruits.

A suggested sequence for training might consider, as a first step, the analysis of deterministic models, with a particular attention to the replacement curves and overcompensation effects. Allee effects and time lags may also be included. Next step might consider deterministic seasonality or periodicity in *R*, by given a set of values of *R* that are applied in sequence. Finally, the effect of stochasticity may be evaluated. The way in which stochasticity is included in the models may have relevant consequences in the future of the simulated populations (discussion in Lande et al., 2003). There are two different sources of stochasticity of *R*: environmental and demographic stochasticity. Sampling error is not considered in this module.

### 2.1 Stochasticity in R

A realistic simulation would require, in a first step, the identification of an environmental variable with a significant effect on the population growth rate; then, the description of a linking function between the environmental variable and the vital rate; and finally, the generation of a sequence of environmental values that mimic natural variability (Caswell, 2001). The sequence of vital rates that will be used to project the population might be then be obtained. No explicit environmental variable, however, is generated by the program. Instead, a generic standardized environmental variable is created. This generic variable is then used to obtain a sequence of population growth rates. The environmental variable may be sampled from three alternative distributions of possible values: a lognormal, a uniform, or a triangular probability distribution. The lognormal distribution is a very well‐known statistical distribution used very often in population models as it is a strictly positive distribution (e.g., Lande, 2002). The uniform and the triangular are both guess distributions and may be used when little information on the shape or nature of the distribution is available. The uniform distribution is adequate when complete uncertainty exists and the triangular when the minimum, the most likely, and the maximum values are known or guessed (Johnson & Kotz, 1999; Manem et al., 2015). The effective population growth rates would follow the same distributions. The users decide which distribution is more adequate for their particular interests.

At the beginning of each projection interval, the application obtains a deterministic population growth rate (*R_det_*). This is the rate at which the population should grow after correcting for density dependence. For the exponential or the ceiling population models, *R_det_* is the default *R* given as input. Evaluation of density dependence includes both, positive (i.e., Allee effects) and negative effects. The second step modifies *R_det_* to accommodate the environmental and demographic stochasticity. The application obtains a distribution of possible values around *R_det_* and an effective growth rate (*R_eff_*) is extracted from that distribution. The mean of such a distribution is the deterministic rate (*R_det_*), and its variance is calculated considering the environmental and the demographic variances:

(5) $$\sigma_{R}^{2}=\sigma_{e}^{2}+\sigma_{d}^{2}/N$$

(see Legendre, 2020, for a recent description). Demographic stochasticity acts independently on individuals and therefore tends to be irrelevant in large populations, as rates average out (e.g., Lande, 2002). The exact *R_eff_* value is extracted differently depending on the nature of the distribution. The exact equations used by the application are in Appendix 1.

Note that by using Equation (5), the effect of demographic stochasticity is forced to be influenced by population size. In other words, the different effects of demographic and environmental stochasticity to appear in the unstructured module are forced by the programing code. This artifact does not occur in the Structured module as the stochastic nature of vital rates is directly implemented by sampling vital rates from a feasible probability distribution (e.g., binomial for survivals).

An alternative way to include stochasticity in the simulations is by sampling a discrete distribution of possible values of the population growth rate, in a similar way as Åberg (1992a, 1992b) did using transition matrices, one matrix for each distinct set of environmental conditions. This may be the only option when the only available information on a population is a limited collection of observed values of *R*. At each projection interval, one of the observed *R* values is randomly obtained by sampling a discrete uniform distribution. This is an obvious oversimplification, as it assumes that the observed set or *R* values are a representative sample of possible values of *R* and, therefore, includes every situation the population is going to face during the whole simulation run. Sometimes, however, this is the only possibility to evaluate the future of a population. Due to the strict assumptions and the paucity of data, interpretation must be necessarily cautious. At this respect, students should be aware that using a probability distribution with the mean and variance obtained from a small sample of *R* values is a naïve approach, which can lead to severely biased results and interpretations: Large confidence intervals are expected for mean and variances for small sample sizes of *R*.

Environmental temporal correlation has a considerable influence on the output of the simulations (e.g., Tuljapurkar & Haridas, 2006). Temporal positive autocorrelation of environments may generate sequences of favorable or unfavorable periods. It has been observed that, on the long run, the variance in final population sizes increases as it does the probability of extinction, though opposite effects may also occur (Ruokolainen et al., 2009). Intuition suggests that the likelihood of series of consecutive projection intervals with unfavorable environments increases as the correlation coefficient increases, increasing also the probability of population extinction. On the other hand, negative autocorrelation in *R* reduces the variance in final populations sizes (Lande et al., 2003) and also the probability of extinction, as extreme years are almost immediately compensated by years with opposite environmental conditions. Consequences of temporal autocorrelation, however, are far from be so simple, and so extinction risk has been shown to increase, but also to decrease, in positively correlated environments (Heino et al., 2000; Schwager et al., 2006). Temporal autocorrelation of temperatures has been recently suggested to increase in scenarios of global change (Cecco & Gouhier, 2018).

**FIGURE 1:**
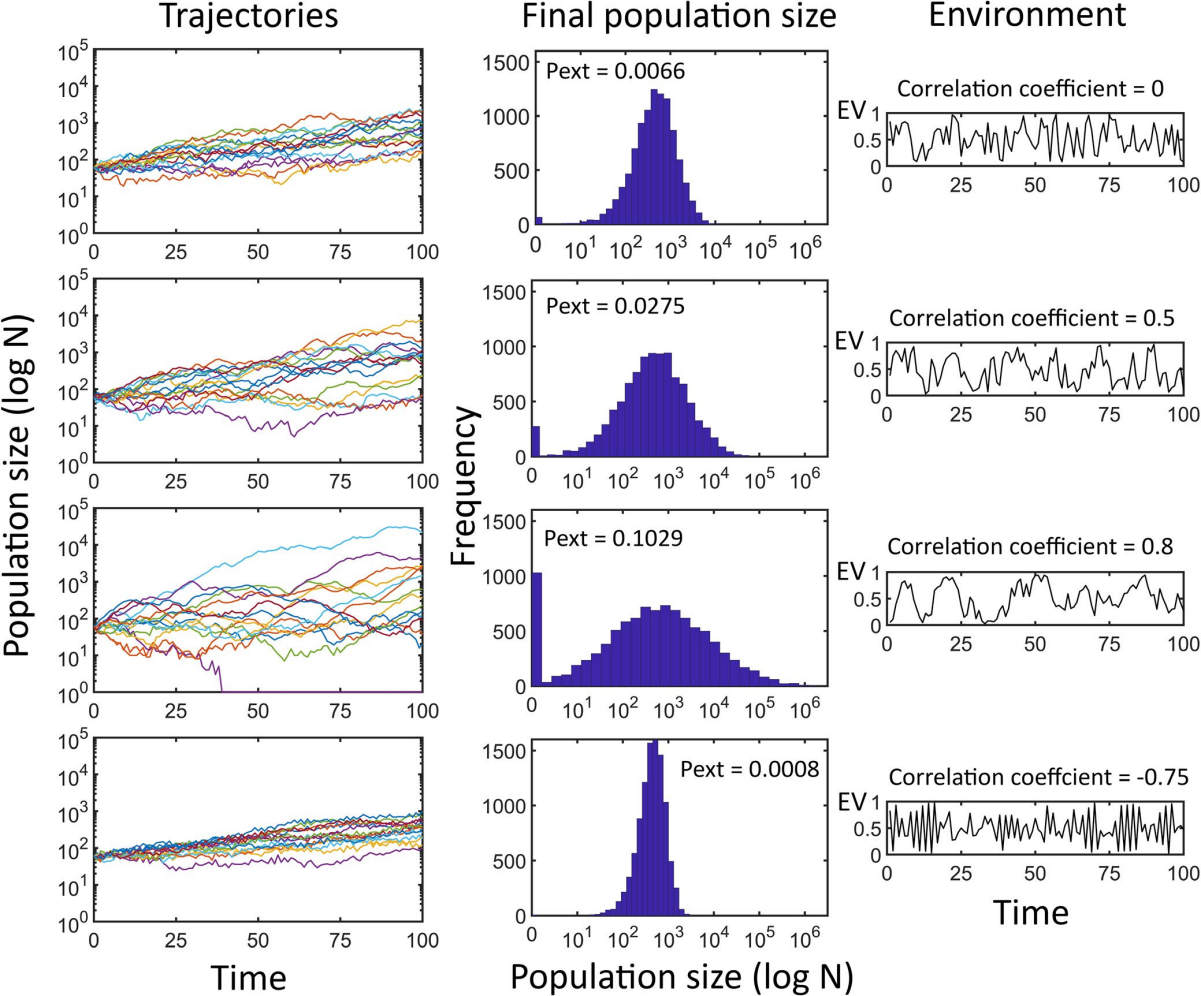
Effect of temporal correlation of environments. Four different simulation runs were done for a population of *Chthamalus montagui* (see Appendix 2 for details). Top to bottom are simulations for independent and identically distributed environments (correlation coefficient = 0), two positively correlated environments with different coefficient and for a negative environmental correlation. Note that coefficients were selected unrealistically large to facilitate visualization of the effects. Left panels are for a sample of trajectories, middle panels for the distribution of final population sizes, and right panels are a selection of representative environments randomly generated with different correlation coefficients. *Pext*, probability of extinction; *EV*, environmental value. Although simulations were done using a matrix model, identical effects of temporal correlation are obtained by using unstructured models

To include temporal correlation in environments, the environmental scores are obtained using an autoregressive model of grade 1 (the environment is only affected by the environment one time step before). A detailed description and enumeration of all autoregressive models used by the application is in Appendix 1. The autoregressive models are a modification of the model described by Ranta et al. (2006). An example of the effect of temporal correlation in population trajectories and the probability of extinction is in Figure 1.

### 2.2 Sensitivity analysis

No explicit sensitivity analysis is included in the Unstructured module. Perhaps the only relevant analysis is the evaluation of the sensitivity (or elasticity) of the probability of extinction to changes in demographic parameters or stochasticity details. Sensitivity (*S_PE_*) and elasticity (*E_PE_*) of the probability of extinction (*PE*) to changes in some parameter (*ρ*) may be calculated by hand by evaluating the probability of extinction before and after a small modification of the parameter used in the simulations:

(6) $$S_{\mathrm{PE}}=\left(PE_{\mathrm{orig}}‐PE_{\mathrm{new}}\right)/\left(\rho_{\mathrm{orig}}‐\rho_{\mathrm{new}}\right),$$

(7) $$E_{\mathrm{PE}}=(PE_{\mathrm{orig}}‐PE_{\mathrm{new}})/f\cdot PE_{\mathrm{orig}},$$

where the subscripts *orig* and *new* denote original and new probabilities of extinction and *f* is the fraction by which the parameter is modified. This expression was used by Crowder et al., (1994) to estimate elasticity of the asymptotic population growth rate to changes in lower level vital rates. The parameter might be, for example, the carrying capacity, the demographic variance, or the correlation coefficient. If the probability of extinction is 0 for the original parameter value, its elasticity cannot be calculated, and the probability of reaching some critical density threshold might be used instead.

## 3 STRUCTURED MODELS

The structured population module includes four types of analysis which may be done in sequence: (a) analysis of asymptotic dynamics and deterministic calculations, (b) the construction of confidence intervals for matrix entries and basic demographic parameters, (c) stochastic projections and evaluation of the probability of extinction, and (d) sensitivity analysis. A simulation consists of *N* replicated evaluations of a population during *T* time steps. At the beginning of each replicate, the population is initialized and a projection matrix is selected to implement sampling error. Then, an environmental sequence is obtained (either *iid* or correlated). At the beginning of each projection interval, the matrix is modified to accommodate environmental stochasticity. Density dependence is then evaluated on the environmental modified matrix and, finally, the effect of demographic stochasticity is evaluated. The population is projected one time step. The process continues until the time horizon is reached or the population is extinct. After completing the *N* replicates, the application gives a collection of population trajectories and the probability of reaching the critical thresholds given by the user. More details are in Appendix 1.

### 3.1 Format of input data

The input may be an already constructed, and ready to use, projection matrix, given either as a single projection matrix or, better, as a fecundity and a transition matrix. The latter option should be preferred, as more information can be extracted from the fecundity and transition matrices than from the projection matrix. The fundamental matrix (a matrix with average times spent at each stage by individuals), the net reproductive rate (*R*_0_), and the generation time (Caswell, 2001) can only be estimated if fecundity and transitions matrices are available. An alternative input is the set of lower level parameters. For each stage, they may be survival, probability of promoting to any other stage (including previous stages or classes) and fertility. Entries of the projection matrix, with the exception of survivals in the Leslie matrix, are often a combination of lower level parameters. It is common also for the matrix entries of each stage to share a common lower level parameter (e.g., survival probability). It might be more convenient to analyze the response of the population to changes in lower level parameters than to changes in matrix entries (e.g., Crowder et al., 1994).

The final input format is raw demographic data. A common problem I observe during practical lessons of Population Ecology is students getting stuck with the construction of the projection matrix. This is relevant because the construction of the projection matrix from census data is a critical step demanding careful thinking. Demography_Lab can obtain the projection matrix from raw demographic data. For each individual, during the projection interval, raw data describe its fate in terms of survival and growth and the number of recruits left. For each stage, and from individual fates, the application obtains the probabilities of remaining or moving to other stages and average fecundity and constructs the projection matrix. This speeds up the construction of the models and guarantees a correctly derived matrix; but it is optional, and thus the pedagogical interest of learning how to construct of matrices is kept by the application. Four different types of raw data are accepted by the program. The types of raw data differ in the available information on reproduction. Anonymous reproduction occurs when parents for recruits cannot be identified. The application distinguishes three types of raw data with anonymous reproduction: type I, when reproductive individuals cannot be recognized in the population; type II, when reproductives are recognized but its origin (in postbreeding censuses) or destination (in prebreeding censuses) are unknown; and type III, when origin or destination of reproductives is known. In the fourth type of raw data, parents for every recruit and recruits for each reproductive are known. The distinction of different types of raw data is relevant to obtain the average fecundity for each stage and to implement the bootstrap. The bootstrap is used for the construction of confidence intervals and to include sampling error in the simulations (see Arrontes, 2018).

Other inputs are the initial population size and structure and the information on density dependence. The user must also give some sampling details such as the timing for the collection of the demographic data (before or after reproduction) and how the individuals were selected for the study. These details are needed for the bootstrap to extract confidence intervals and to implement sampling error associated with the construction of the projection matrix (see below). Additional information on input formats is in Arrontes (2018).

### 3.2 Analysis of seasonal matrices

Seasonal matrices describe transitions and fecundity schemes in environments with a cyclic or periodic variation. It may be seasonal or interannual variation. Each season or distinct period has an associated projection matrix. There may be an obvious interest in these models when very distinct vital rates, and therefore management options, are associated with different predictable periods. Seasonal models are also useful, for example, for annual species (Caswell, 2001) and to manage pest species by selecting the most effective period for pest control (e.g., Darwin & Williams, 1964; Smith & Trout, 1994) or for the selection of the optimal hunting or extraction period in game or exploited species (Angulo & Villafuerte, 2003). In these cases, a control matrix is created (a matrix of zeros with proportions or ones in the main diagonal). The analysis of individual periodic matrices is often useless, as these matrices may not be operating on their stable stage structure, but on the structure generated by the previous matrix. This is well illustrated by Vavrek et al. (1997). More relevant is how the dynamics at different periods may influence the annual dynamics. The analysis of the annual matrix may be also misleading. Each entry in the annual matrix is the sum of several terms, each of which is a combination of vital rates from different periods. See additional comments in Caswell and Trevisan (1994). The options of environmental stochasticity are limited for seasonal matrices.

### 3.3 Control of density dependence

Density dependence may be included in two different ways. The simplest is a ceiling‐like approach which automatically adjusts the population size when the population is above some threshold given by the user. The user may specify which ages or stages are affected by, and are responsible for, density dependence. The program regulates the number of individuals in the affected classes before projecting the population one time step. The second approach modifies entries in the projection matrix using a density‐dependent function. The user specifies which matrix entries are affected by density dependence. Then, for each affected vital rate, the user identifies the stages responsible for density dependence. The user also specifies the carrying capacity and the function modifying the rate. Three functions are available; a Ricker (8), a Beverton–Holt (9), (e.g., Turchin, 2003) or a ceiling‐like function (10):

(8) $$a_{\mathrm{ij}}^{*}=a_{\mathrm{ij}}\exp\left(‐cN\right)\;\;\text{with}\;c≃\ln\left(\lambda \right)/K$$

(9) $$a_{\mathrm{ij}}^{*}=a_{\mathrm{ij}}\left[1/\left(1+cN\right)\right]\;\text{with}\;c≃\left(\lambda ‐1\right)/K$$

(10) $$\begin{array}{cc} a_{\mathrm{ij}}^{*}=a_{\mathrm{ij}} & \text{if}\;N\leq K\\ a_{\mathrm{ij}}^{*}=a_{\mathrm{ij}}c/N & \text{if}\;N>K\;\text{with}\;c≃K/\lambda \end{array},$$

where $a_{\mathrm{ij}}^{*}$ and $a_{\mathrm{ij}}$ are, respectively, the modified and the original matrix entries; *N* is the number of individuals in the stages responsible for density dependence. *N* may be the total population size, if all stages are responsible, or the number of individuals in some specific stage, reproductive individuals, for example. λ is the asymptotic population growth rate; and *K* is carrying capacity and is the population size at which the populations should stabilize if all stages and matrix entries were responsible, and affected, by density dependence.

### 3.4 Construction of confidence intervals for basic demographic parameters and matrix entries

Specifically, 90% and 95% confidence intervals are constructed for matrix entries, the asymptotic population growth rate, the stable stage structure, and the reproductive value. To obtain the intervals, the application needs the number of individuals used to construct the projection matrix and how these were sampled from the population (at random or using fixed quotas for each stage). Intervals are obtained using the bootstrap (Efron & Tibshirani, 1993) as described in Caswell (2001). In short, the bootstrap consists in resampling with replacement the sample of individuals originally used to construct the matrix. With the bootstrap sample of individuals, a new projection matrix is obtained (the bootstrap matrix) and demographic parameters are extracted. After obtaining a large number (say 10,000) of bootstrap matrices, the 90% or 95% confidence intervals may be obtained by extracting the 5 and 95 or the 2.5% and 97.5% percentiles, respectively (a more detailed description may be also found in Arrontes, 2018).

### 3.5 Sensitivity analysis

Perturbation analyses done by Demography_Lab include sensitivity and elasticity of the asymptotic population growth rate, *λ*, to changes in matrix entries and also the analysis of the changes in the probability of reaching a threshold in population size. The user may choose to do the sensitivity analysis of *λ* analytically, by using the right and left eigenvectors (the application uses the MATLAB code given in Caswell, 2001), or may evaluate the changes in *λ* in response to changes in vital rates by using variations of equations (6) and (7) (as in Crowder et al., 1994). Another indirect way to estimate sensitivity of *λ* is to obtain the range of possible values of *λ* in response to the range of possible values of selected vital rates. This gives an estimation of the uncertainty in *λ* values in response to uncertainty in vital rates (Akçakaya et al., 1999). The evaluation of the probability of reaching a size threshold includes the probability of extinction and the probability of reaching some safe population size. The probability of reaching a size threshold is evaluated in response to changes in vital rates, to changes in the intensity of density dependence, and to changes in the magnitude of the environmental stochasticity.

### 3.6 Stochasticity options

**FIGURE 2:**
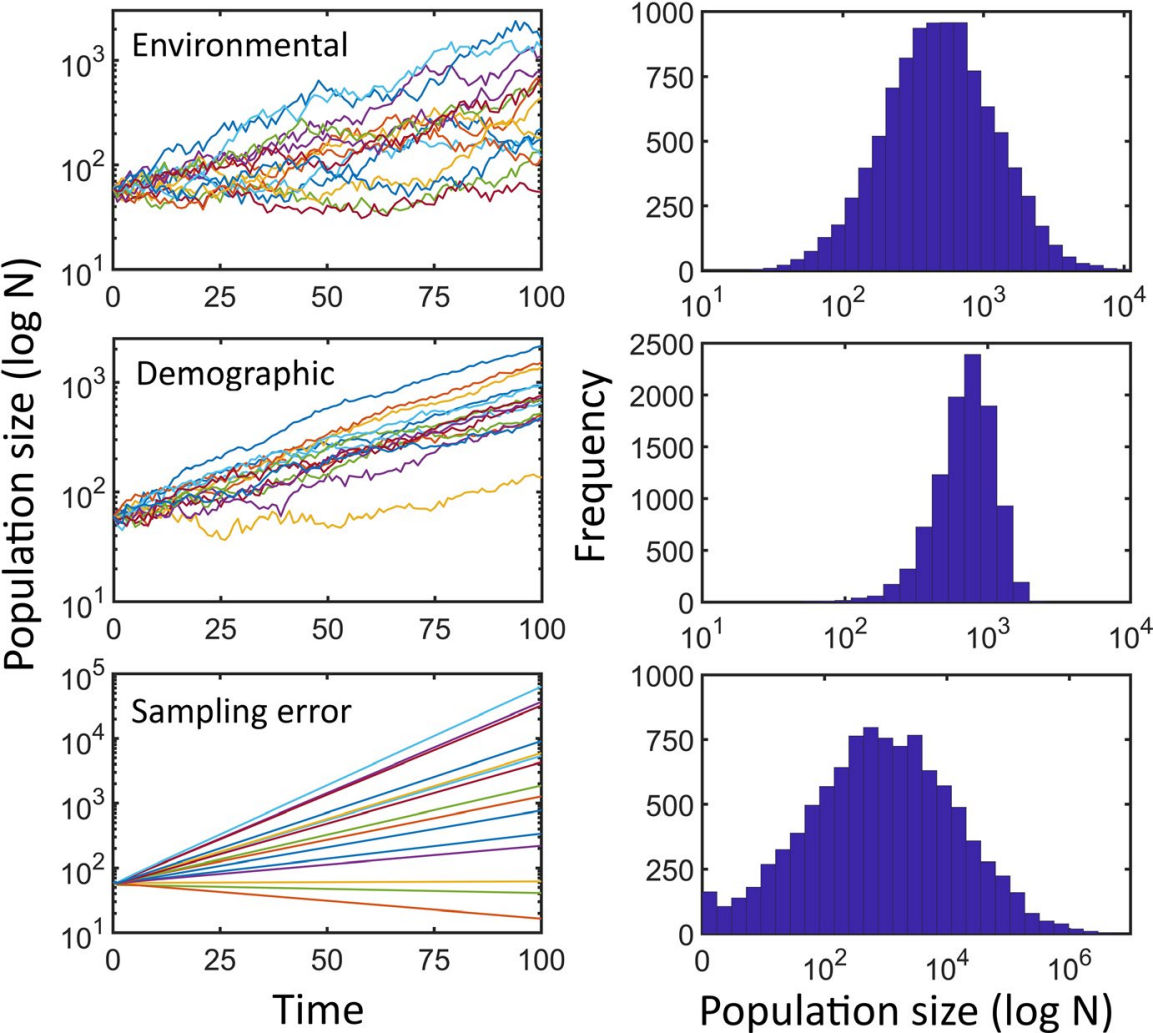
Effect of different sources of stochasticity on simulated population trajectories for a hypothetical population of the barnacle *Chthamalus montagui* (see Appendix 2 for details). Three independent runs were done, with a single source of stochasticity in each: only environmental stochasticity (environmental, top panel), only demographic stochasticity (demographic), and only sampling error. Because sampling error is the only source of stochasticity, and it is implemented by selecting a different projection matrix for the whole replicate, trajectories are deterministic. Left panels are the 15 first replicated trajectories. The right panels are for the distribution of final population sizes for the 10,000 replicates. Note the different scales

The application evaluates populations under three sources of stochasticity: environmental, demographic, and error in the construction of the projection matrix (Lande et al., 2003). The two former sources are real processes affecting populations. The latter does not affect real populations but it may severely affect our calculations or simulations. See Figure 2 for an example of the effect of the different sources of stochasticity. The application offers different options to implement each source of stochasticity.

Environmental stochasticity may be included in three ways. Most simple is by sampling a discrete set of matrices (Åberg, 1992a, 1992b; see also Nakaoka, 1996), each representing an environmental stage. This is the “random transmission matrix” approach of Fieberg and Ellner (2001). The set of possible matrices may be the matrices observed during some period of study involving several projection intervals; or may be matrices associated with contrasting environmental conditions. A second option is to obtain vital rates in the projection matrix after sampling a range of possible values given by the user. This method includes the selection of the probability distribution associated with the vital rates (normal or uniform) and the degree of correlation among vital rates in response to environmental variability. This is equivalent to sampling a multivariate distribution describing the variation of vital rates under the “parametric matrix method” approach of Fieberg and Ellner (2001) and is how some software packages work, such as Ramas or Vortex (Akçakaya et al., 1999; Lacy & Pollak, 2020). A third option used in Demography_Lab obtains the projection matrix after sampling vital rates using a matrix of variance–covariance among vital rates. This is another example of the previous approach. At each, projection interval, the application obtains a sample of vital rates from a multivariate normal or uniform distribution constrained by the variance–covariance matrix given by the user.

The effect of the environmental variability depends both on the magnitude of the environmental changes and on the structure of the projection matrix. Negative correlations among vital rates may substantially reduce the effect of environmental variations (Doak, Morris, et al., 2005; Tuljapurkar, 1990). The user has control on the correlation among matrix entries. The application also offers the possibility to fix an environmental sequence. Differences among runs depend only on the effects of demographic stochasticity and, if applicable, to the effects sampling error in the construction of the projection matrix. Obviously, this has just a pedagogical interest, as it helps to identify the relevance of demographic stochasticity and sampling error. As for the unstructured module, temporal correlation of environments is explicitly included and it is under full control by the user (see Appendix 1).

Catastrophes are not considered by the application. Catastrophes are unpredictable environmental events, causing a substantial reduction in population size. They can be considered an additional source of stochasticity to populations (e.g., Shaffer, 1981; Young, 1994) or an extreme case of environmental stochasticity (Lande et al., 2003; Shaffer, 1987). There are not objective reasons to ignore catastrophes and they might be included in future improvements of the application.

Demographic stochasticity is evaluated separately for transitions and fecundities. The user may select the vital rates affected by demographic stochasticity. Demographic stochasticity is evaluated after sampling error, environmental stochasticity, and density dependence have been evaluated. The magnitude of the effect of demographic stochasticity depends on the number of individuals at each class, and therefore is expected to change as population size changes during the simulations. Demographic stochasticity is a magnitude difficult to estimate. In simple models, demographic stochasticity might be included as deviations in the expected number of individuals surviving the projection interval and in the number of recruits left by reproductive individual. Deviations in survival may be quantified as the variance of a binomial distribution (Kendall & Fox, 2002; Legendre, 2020). Variance associated with the recruits should be related to the variance of the probability distribution of the reproductive output of individuals (Akçakaya et al., 1999; Kendall & Fox, 2002). The application offers three possibilities, Poisson, uniform, and a discrete user‐given distributions. The effect of demographic stochasticity on growth is also evaluated by sampling binomial or multinomial distributions. To speed up simulations at runtime, demographic stochasticity is evaluated only for classes in which the number of individuals is larger than 4,000. This is not expected to alter the output, because at large densities, demographic stochasticity is almost irrelevant.

**FIGURE 3:**
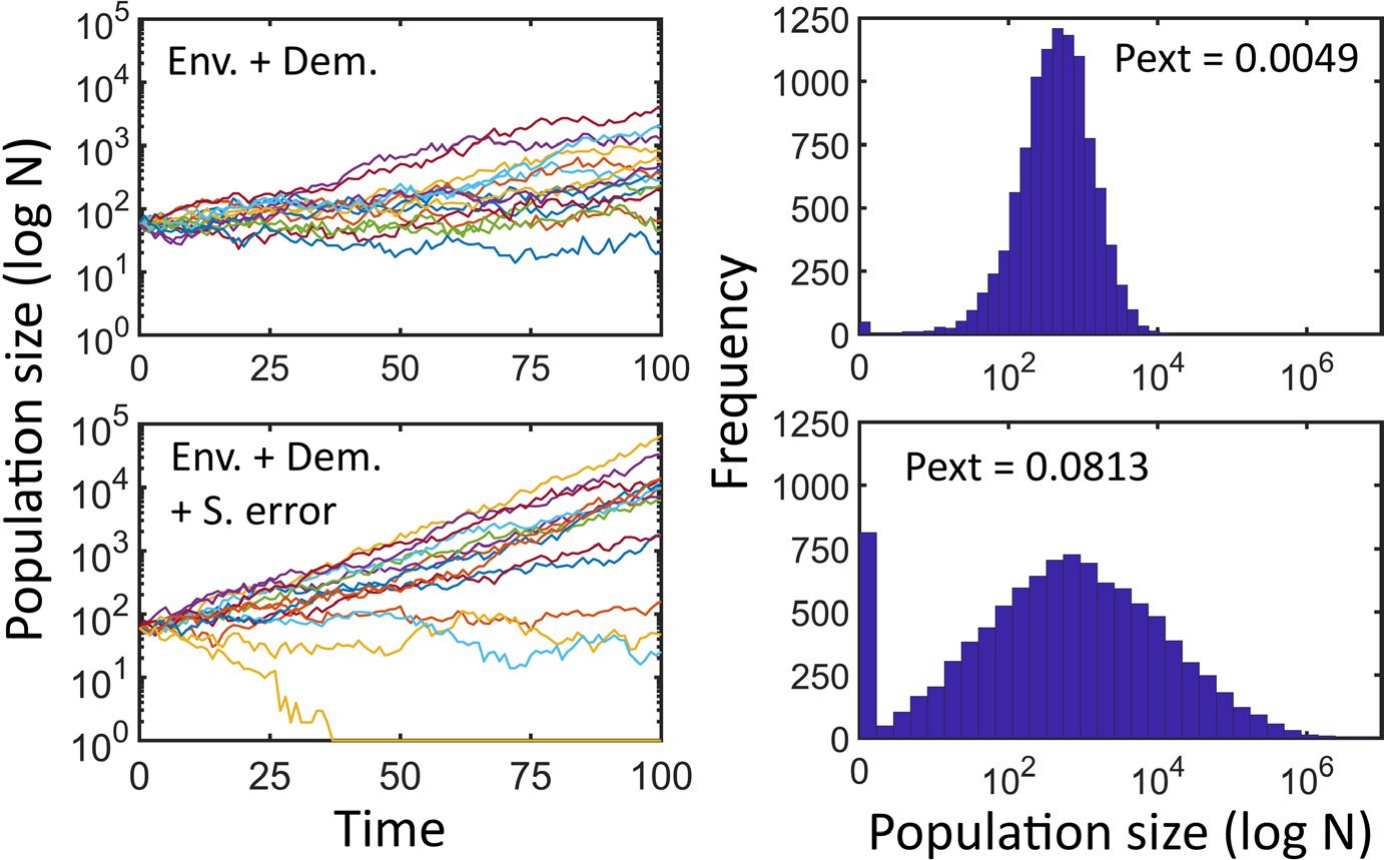
The effect of sampling error on the output of a stochastic matrix model for a population of *Chthamalus montagui* (see Appendix 2 for details). *Env + Dem*, top panels, are for a simulation run using environmental and demographic stochasticity. *Env* + *Dem* + *S error*, bottom panels, are for a simulation run in which sampling error is included. Sampling error was implemented by using the bootstrap. For each of the 10,000 realizations of the model, a new projection matrix was obtained after extracting a bootstrap sample of the individuals used to construct the original projection matrix. Left panels show a sample of 15 trajectories. Right panels show the distribution of final population sizes. *Pext*, probability of extinction

Sampling error is associated with the uncertainty in the construction of the projection matrix. Sampling error is a consequence of the finite number of individuals used (or sampled) to estimate vital rates. As for any other sampling program, small number of individuals leads to wide confidence intervals for vital rates (McCallum, 2000). This means that very distinct matrices might be equally possible, which increases the variability of the future trajectories of the population (Figure 3). Sampling error increases the range of final population sizes and the probability of extinction. To implement sampling error, matrices are randomly obtained by the application before each simulation run. The user may choose from two alternative methods: by using the bootstrap, which involves resampling the sample of individuals used to construct the projection matrix; or by sampling vital rates from a range of possible values, given by the user.

## 4 USING DEMOGRAPHY_LAB AS A TEACHING TOOL

No formal tests were done on the performance of the students before and after using the application. However, after using the application for several years, a few conclusions may be obtained. The performance of undergraduate students is better using Demography‐Lab that using general purpose software (like R or MATLAB). Of course, mastering general purpose software for modeling and specialized applications (such as Vortex, Lacy & Pollak, 2020) is part of the teaching objectives of advanced Applied Population Ecology courses. But for introductory courses, paraphrasing Burch (2018, p. 157), the emphasis of mathematical modeling in Demography should not be “so much on the rigor of quantifications as on the ability to perform complex logical inferences correctly.”

Teaching was also benefited by the scalable nature of the application. Using the same application through the different levels of learning implies that students are familiar with the user interface, operational aspects and the nature and interpretation of the outputs. Savings in time and effort were not quantified, but were evident. Note that scalability is considerable, since Demography‐Lab has two other advanced modules to evaluate metapopulation and individual based models (not presented in this report), and an additional module to evaluate statistical differences among population parameters (Arrontes, 2018).

By using Demography_Lab, the students go through the whole process of constructing a population model as suggested by Legendre (2020), from the analysis of the life cycle of the species and extraction of demographic information to sensitivity analysis. Students receive three important messages. First, that variability in vital rates decreases future grow in populations. Second, that in scenarios of global change, increases in variance might be as influential (or more) as changes in average values of vital rates (Vasseur et al., 2014). And third, that small population size may be responsible for populations entering an extinction vortex (Lande, 2002). At this respect, students realize that in declining populations, demographic variance becomes a relevant source of stochasticity affecting the population growth rate. By evaluating demographic and environmental stochasticity separately, students gain insights into the different effects and relevance of both sources of variability.

Students learn that the construction of realistic and reliable models may ask for considerable demographic and environmental data. But also, that population models may be constructed with very limited and incomplete demographic information. At this respect, sensitivity analysis helps to identify how robust a model is against small changes in input parameters; particularly against the expected noise in vital rates associated with sampling error. Students may experience one of the axioms of modeling, that even though simulation is possible with limited data, the quality of the outputs depends on the quality of the inputs.

## CONFLICT OF INTEREST

None declared.

## AUTHOR CONTRIBUTION

**Julio Arrontes:** Conceptualization (equal); data curation (equal); formal analysis (equal); funding acquisition (equal); investigation (equal); methodology (equal); resources (equal); software (equal); validation (equal); visualization (equal); writing – original draft (equal); writing – review and editing (equal).

## Figures and Tables

**FIGURE A1:**
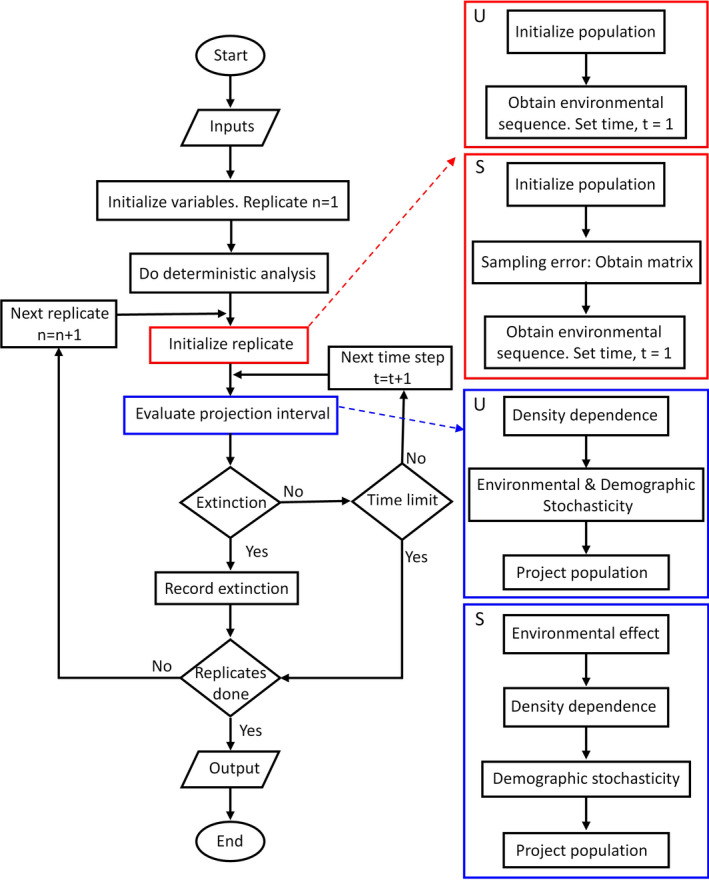
Flowchart for Demography_Lab. **U,** steps for the Unstructured module. **S**, steps for the Structured module. Depending on the conditions of the simulation specified by the user, some of the steps may be ignored

## Data Availability

No original data were collected for this manuscript. The application, and a detailed description, may be accessed from the Open Science Framework at https://osf.io/rn4t3/.

## APPENDIX 1. COMPUTATIONAL DETAILS FOR Demography_Lab

A simulation consists of *N* replicated evaluations of a population during *T* time steps. At each replicate and time step, the projection matrix given as input is modified to accommodate stochasticity and density dependence. A flowchart for a simulation run is in Figure A1. The output is the complete set of trajectories (*N* replicates and *T* time steps in each) and the probability of reaching low and high size thresholds. Reading of inputs may include (a) demographic data within a variety of formats and the initial population size and structure; (b) details for density dependence; (c) stochasticity details: sampling error, environmental stochasticity, temporal correlation of environments, and demographic stochasticity; and (d) simulation details: number of replicates, number of time steps, and density thresholds.

The application has a limited offer of probability distributions for demographic parameters and environmental and demographic stochasticity. Some realistic probability distributions in demography are left out, for example, the negative binomial or the Gamma distributions (Otto & Day, 2007). This is not a problem for the main objective of the application, training in demography, which can be covered with well‐known distributions to the students as the uniform or the normal and lognormal distributions.

### IMPLEMENTING STOCHASTICITY IN THE UNSTRUCTURED MODULE

A preliminary comment illustrates that the selection of the model may not be trivial and may have profound implications in the output. The use of deterministic models as a base to construct stochastic models may lead to significant artifacts during simulations. For example, if the Ricker or the Beverton–Holt models are used, under particular conditions they may predict a population explosion. The artifact occurs when *R* < 1 and the population size is above carrying capacity; if *R* < 1 and *N_t_ > K*, then $R^{\left(1‐N_{t}/K\right)}>1$, and the populations explodes to infinity. This scenario should be impossible in a deterministic world, as a population with *R* < 1 should never be above *K*, but it may occur under stochastic environments.

#### Lognormal distribution of environments

For the lognormal distribution, the effective population growth rate is extracted from the log‐transformed rates (see, e.g., Lande et al., 2003). In a log scale, the population growth rate follows a normal distribution with mean

(A1) $$\bar{r}=\ln\bar{R}+\sigma_{r}^{2}/2$$

and variance

(A2) $$\sigma_{r}^{2}=\ln\left(1+\sigma_{R}^{2}/{\bar{R}}^{2}\right),$$

The variance of the deterministic rate is calculated considering the environmental and the demographic variances:

(A3) $$\sigma_{R}^{2}=\sigma_{e}^{2}+\sigma_{d}^{2}/N$$

The effective rate is extracted by transforming an environmental score (*e_t_*) randomly obtained from a *N*(0, 1). The distribution is truncated at *p* = 0.001 to avoid extreme, and perhaps unrealistic, values. The environmental score is converted to an *r_eff_* value:

(A4) $$r_{\mathrm{eff}}=\bar{r}+e_{t}\sigma_{r}$$

and then transformed to the linear scale: $R_{\mathrm{eff}}=\exp\left(r_{\mathrm{eff}}\right)$.

For an independent and identically distributed (*iid*) environment, the score is extracted at the beginning of each projection interval. Scores for correlated environments are always obtained by using a simple autoregressive model:

(A5) $$e_{t}=\left(1‐\kappa \right)\bar{e}+\kappa e_{t‐1}+SD\sqrt{1‐\kappa^{2}}\varepsilon_{t}$$

where *e_t_*, *e_t_*_−1_ are environmental deviates for times *t* and *t* − 1, $\bar{e}$ is the average environmental value, *κ* is the correlation coefficient, *SD* is the standard deviation of the normal deviate (for Gaussian noise) or the range of the distribution (for uniform noise), and *ε_t_* is a normal deviate randomly obtained from a normal distribution *N*(0,1) (for Gaussian noise) or a uniform deviate obtained from a uniform distribution (0,1) (uniform noise).

Because the environments are created using a *N*(0,1) distribution, the autoregressive model simplifies to:

(A6) $$e_{t}=\kappa e_{t‐1}+z_{t}\sqrt{1‐\kappa^{2}},$$

where *z_t_* is a *N*(0, 1) deviate and other elements are as in Equation (A5).

#### Uniform or triangular distribution of environments

For uniform and triangular distributions, *R_eff_* is extracted after randomly sampling a uniform standard distribution *U*(0, 1) representing the environmental value of the year. *R_eff_* is obtained by applying inverse methods to the probability represented by the uniform deviate. From the relationship between range and variance, $\sigma_{R}^{2}=range^{2}/12$ and $\sigma_{R}^{2}=range^{2}/24$, for the uniform and triangular distributions respectively, a minimum and maximum deterministic values for *R* are calculated.

For the uniform distribution, and an *iid* environment, *R_eff_* is calculated as:

(A7) $$R_{\mathrm{eff}}=minR+e_{t}\cdot range.$$

For the triangular distribution:

(A8) $$\begin{array}{cc} R_{\mathrm{eff}}=minR+range\sqrt{e_{t}/2} & \text{if}\;e_{t}\leq 0.5\\ R_{\mathrm{eff}}=maxR‐range\sqrt{(1‐e_{t})/2} & \text{if}\;e_{t}>0.5 \end{array},$$

If environment is *iid*, *e_t_ = u_t_*, random deviate from a uniform *U*(0, 1).

If environments are correlated:

(A9) $$e_{t}=\left(1‐\kappa \right)0.5+\kappa e_{t‐1}+\left(u_{t}‐0.5\right)\sqrt{1‐\kappa^{2}},$$

where *u_t_* is a *U*(0,1) deviate and other elements in the equation are as in Equation (A5).

#### A note on demographic variance

Demographic variance may be given directly by the user or may be calculated by the application from the population growth rate (*R*). The procedure is identical to that used by Akçakaya et al. (1999) in Ramas Ecolab. Briefly, *R* is the sum of average survival probability of individuals and the average number of recruits they left, *R* = *s* + *m*. In the absence of any other source of stochasticity, variance of *R* should be only due to the stochastic nature of the processes responsible for *R* (survival and fertility). The application assumes that survival and reproduction are independent, that survival follows a binomial distribution, with mean *s* and variance *s*(1 − *s*), and that the number of recruits follows a Poisson distribution, with mean and variance *m*. The user only gives the value of average survival (*s*). The demographic variance can then be estimated as:

(A10) $$\sigma_{d}^{2}=\sigma_{R}^{2}=s\left(1‐s\right)+m$$

### IMPLEMENTING STOCHASTICITY IN THE STRUCTURED MODULE

#### Environmental stochasticity

##### A set of possible alternative matrices

The user enters a set of possible projection matrices and their occurrence probability. For *iid* environments, an environmental value from a *U*(0,1) distribution is randomly extracted and then, using inverse methods, a multinomial distribution is sampled. For correlated environments, the autoregressive model is used: The environment is created by sampling a uniform, *U*(0,1), distribution. The autoregressive model is identical to Equation (A9) above. Then, to extract the appropriate matrix for each projection interval, a multinomial distribution is sampled by using inverse methods.

##### A matrix with ranges of vital rates

The user enters a matrix with the ranges of possible values for each vital rate in the projection matrix. The user must also specify the correlation among the vital rates. Any correlation is possible, from identical response of every vital rate to environmental variation to independent effect of the environment on vital rates. These two extremes are unlikely and the most common situation should be some intermediate value (default: 0.8). To generate sets of vital rates with a given correlation, a correlation matrix among vital rates is created. This is a square matrix (its dimension being the number of nonzero vital rates) in which the main diagonal are ones and any other entry is the correlation coefficient. The next step is to obtain at random, and independently, a *N*(0, 1) deviate for each of the vital rates. The set of correlated values (***e*** *_t_*) is obtained from

(A11) $$e_{t}=z_{t}C$$

where ***z_t_*** is a row vector with the set of independent *N*(0, 1) deviates and ***C*** is the Cholesky‐like decomposition of the correlation matrix. At this step, we have a set of correlated normal *N*(0, 1) deviates representing the environmental effect on each of the vital rates in the projection matrix.

By chance, it may occur that some of the normal deviates are beyond the truncation limits given by the user (e.g., [−1.96, 1.96], leaving out 2.5% of the values at each side of the distribution). The out‐of‐range normal deviate is then wrapped by using the expression:

(A12) Wrapped value=2×limit‐observed value.

The limits behave as bouncing boundaries.

The conversion of environmental values into vital rates depends on the distribution of values within the interval. If the probability distribution for vital rates is normal, *N*(0, 1) deviates can be transformed into a vital rate value as: *Vital rate = mean + SD* × *deviate*. At this stage, the projection matrix is ready. The standard deviation (*SD*) is obtained from the ranges given by the user:

(A13) $$SD=\frac{min‐mean}{z_{\min}}$$

*z_min_* is the lower *z*‐score value associated with the truncation value given by the user; *min* is the lower limit of the range of the possible values for the vital rate; and *mean* is the vital rate entered by the user as the average projection matrix.

Any normal deviate which is out of range is wrapped as described above and then, vital rates are extracted from the input ranges and the projection matrix obtained.

In correlated environments, for environments created by sampling a standard normal distribution, *N*(0,1), the sets of vital rates are correlated. The environment for a single year is a collection of random normal deviates; one for each nonzero entry in the projection matrix. The standard deviation of the random deviate in the stochastic component of the autoregressive model is replaced by the Cholesky‐like decomposition of the correlation matrix. The autoregressive model becomes:

(A14) $$e_{t}=\kappa e_{t‐1}+z_{t}C\sqrt{1‐\kappa^{2}}$$

where ***e*** *_t_*, ***e*** *_t_*_−1_ are row vectors with environmental values for each vital rate at times *t* and *t* − 1; *κ* is the temporal correlation coefficient; ***z*** *_t_* is a row vector with random and independently obtained *N*(0, 1) deviates at time *t*; and ***C*** is the matrix with the Cholesky‐like decomposition of the correlation matrix (correlation among vital rates).

If the probability distribution for the vital rates is uniform, the set of normal deviates are transformed into probabilities. The transformation is done after the set of correlated deviates have been obtained and extreme values wrapped. The obtained probabilities are equivalent to deviates from a standard uniform distribution, *U*(0, 1), with 0 and 1 being the minimum and maximum values. The *U*(0, 1) deviates are then transformed into vital rates as: *Vital rate = min + range* × *deviate,* where the *min* value is obtained as before.

##### A matrix with covariances of vital rates

Environmental stochasticity is implemented by sampling a multivariate distribution of vital rates whose mean is the matrix entered as input and its variance is defined by a variance–covariance matrix of vital rates. At each projection interval, a matrix is obtained from this multivariate distribution (actually, what is obtained is a set of vital rates). The covariance matrix may be entered directly by the user or it may be created by the application. To create the covariance matrix, the user must give a set of possible projection matrices: Then, the application calculates the variances and covariances of the vital rates.

Irrespective of the probability distribution of the vital rates, the new projection matrix for each period is always obtained by randomly sampling a multivariate normal distribution. The mean of such a distribution is the original projection matrix entered as input. A row vector, with random and independently obtained normal, *N*(0, 1), deviates is obtained. The length of the vector is the number of nonzero entries in the average projection matrix (i.e., the number of vital rates). To generate matrices with the specified pattern of variance–covariances among vital rates, the Cholesky‐like decomposition of the variance–covariance matrix is used. The vector with the vital rates to reconstruct the projection matrix to be used at time *t* is obtained as:

(A15) $$a_{t}=a_{m}+z_{t}C$$

where ***a_t_*** is a row vector with the vital rates at time *t*; ***a_m_*** is a row vector with the mean vital rates (as entered in the projection matrix used as input); ***z_t_*** is the row vector with independent normal deviates; and ***C*** is the Cholesky decomposition of the variance–covariance matrix.

The projection matrices are reconstructed from the row vectors with the vital rates (***a_t_***). If the probability distribution of the values of the vital rates is normal, no additional transformation is needed. The projection matrix is constructed by substituting nonzero elements by the elements in the row vector.

###### Correlated environments

Environments are created by sampling a standard Normal distribution, *N*(0,1). The environment for a single year is a collection of random normal deviates; one for each nonzero entry in the projection matrix. The standard deviation of the random deviate in the stochastic component of the autoregressive model is replaced by the Cholesky‐like decomposition of the variance–covariance matrix (given as input by the user). Instead of environmental values or indices, the autoregressive model gives the final vital rates and becomes:

(A16) $$a_{t}=\left(1‐\kappa \right)a_{m}+\kappa a_{t‐1}+z_{t}C\sqrt{1‐\kappa^{2}},$$

where ***a_t_***, ***a_t_***_−_ ***_1_*** are row vectors with the vital rates at times *t* and *t* − 1; *κ* is the temporal correlation coefficient; ***a_m_*** is a row vector with the mean vital rates (entries of the matrix used as input); ***z_t_*** is the row vector with independently obtained normal deviates; and ***C*** is the Cholesky decomposition of the variance–covariance matrix.

###### Uniform distribution

If the probability distribution of the vital rates is uniform, a modification is needed: The ***a_t_*** vector is converted into a ***u_t_*** vector with vital rates extracted from a uniform distribution. As a first step, the vital rates in ***a_t_*** must be transformed into probabilities using the normal cumulative distribution. The ranges of the uniform distributions of the vital rates are calculated from the variances of vital rates given by the user as: $r_{\mathrm{ij}}=\sqrt{12\times \sigma_{\mathrm{ij}}^{2}}$, for the vital rate at row *i* and column *j*. The vector with the vital rates for period *t* is then obtained as:

(A17) $$u_{t}=\left(a_{m}‐r/2\right)+r\circ p_{t},$$

where ○ denotes the Hadamard product of two matrices (or element‐by‐element multiplication) and ***u_t_*** is a row vector with the vital rates at time *t*; ***a_m_*** is a row vector with the mean vital rates (entries of the matrix used as input); ***r*** is a row vector with the ranges for each nonzero vital rate; and ***p_t_*** is a row vector with probabilities associated with each vital rate at time *t*.

#### Demographic stochasticity

##### Transitions (survival and growth)

If low level vital rates are used as input data, survival and growth of individuals are evaluated independently. Survival is evaluated by sampling a binomial distribution *B*(*N_i_*, *S_i_*), where *N_i_* is the number of individuals at stage *i* at the beginning of the interval and *S_i_* is the survival probability during the interval. For individuals surviving, the destination stage (growth) is evaluated by sampling a multinomial distribution describing the probability of remaining in the stage or ending in any other stage. If the census is prebreeding, survival of recruits (or newborns) is evaluated separately. It is done by sampling a binomial distribution *B*(*Nr*, *Pr*), where *Nr* is the expected total number of recruits produced during the reproductive period and *Pr* is the survival probability of recruits during the projection interval.

For any other input format, demographic stochasticity is evaluated on the whole projection matrix or on fecundity + transitions matrices. Destination of the individuals at each stage is evaluated by sampling a multinomial distribution. For stages with a single destination (in addition to death), a binomial distribution is sampled.

##### Fecundities and fertilities

Fertility is the average number of newborns produced by reproductive individual during the reproductive season. It is related to the biological potential to produce new individuals. Fecundity is the realized fertility during the projection interval. It is the average number of new individuals in the population produced by reproductive individuals during the projection interval. It is a combination of fertility and survival. If input data are low level vital rates, fertility is used to evaluate demographic stochasticity related to reproduction. For all other input formats, fecundity is used. In both cases, demographic stochasticity considers the probability distribution of the numbers of recruits or newborns. Three probability distributions are used: Poisson, uniform, and discrete, user‐given, distributions. Hereafter, only the term fecundity is used.

###### Poisson distribution

This probability distribution is discrete and has a single parameter, (commonly represented by *λ*, which has nothing to see with the asymptotic population growth rate*)*, which is both the mean and variance of the distribution. For each stage, *λ* is the average fecundity of the stage. To evaluate demographic stochasticity, for individuals at each stage *i*, a Poisson distribution with *λ_i_* identical to fecundity of the stage, is randomly sampled *N_i_* times, where *N_i_* is the number of individuals in the stage. As fecundity changes as consequence of environmental stochasticity or density dependence, new Poisson distributions with the new *λ_i's_* are sampled.

###### Uniform distribution

The user gives a range of possible values around the average fecundity in the projection matrix. The number of recruits produced by the individuals at each stage during the projection interval is obtained by sampling the uniform distribution associated with each stage as many times as individuals there are in the stage at the beginning of the projection interval. After the fecundity is modified by the environment or density dependence, the range of new uniform distributions keeps unaltered. If the minimum value becomes negative under very low fecundities, the range is adjusted to have 0 as minimum value. The maximum value is adjusted to keep the new fecundity as the mean of the distribution.

###### Discrete distribution

The user must enter the discrete probability distribution in the form of pairs of values [number of new individuals produced, probability] for each reproductive stage. The number of recruits is obtained by random sampling of a multinomial probability distribution as many times as individuals are in the reproductive stages. There is a problem with this approach, as the original user‐given distribution should only be valid for the original projection matrix. After the modification of the projection matrix by environmental stochasticity and density dependence, the user‐given distributions should be changed, as fecundities are modified. There is not an automatic and easy way to adjust the discrete distribution to the new average fecundity. The application keeps constant the number of classes and the probabilities associated with each class. The fecundities, however, are modified by the same factor the average fecundity was changed in the projection matrix. This approach necessarily assumes that the individuals may leave fractional numbers of newborns. I ignore the effect of the resulting stochasticity but I presume that with this approach demographic stochasticity might be only slightly underestimated.

#### Sampling error in obtaining the matrix

##### Using a bootstrap procedure

The user must introduce information on how demographic data were obtained. These include (a) the number of individuals studied in each stage and from which the projection matrix was constructed; (b) if individuals were selected at random or if a fixed numbers of individuals were studied in each stage, and (c) the identification of reproductive stages in postbreeding censuses.

The first step in the bootstrap is the construction of an auxiliary matrix with the individual histories during the projection interval. The auxiliary matrix contains all the information needed to reconstruct the projection matrix. Each column in the auxiliary matrix is an individual during the projection interval. The first row defines the stage at the start of the projection interval. The second row, the destination stage (including death). The final row(s) give the number of recruits left by the individual. There should be as many rows as classes to which new individuals recruit. Details for the shape and construction of the auxiliary matrix have been explained elsewhere (Arrontes, 2018).

Random sampling of individual histories with replacement from the auxiliary matrix simulates repeated sampling of the original population in the field, and so different projection matrices may be obtained. At the start of each simulation run, the application obtains a random sample of individual histories and extracts the projection matrix. The random sample is obtained by sampling, with replacement, a discrete uniform distribution *U*(1, *N*) (Caswell, 2001). The sample size, *N*, is the original sample size used to construct the original input matrix and is the length of the auxiliary matrix. If sample size was originally large, differences among randomly extracted matrices will be small and the variability due to sampling error should be small. If the original sample size was small, matrices may differ considerably and the sampling error should be large.

The procedure is not affected by the nature of the environmental stochasticity. However, if the environmental stochasticity is implemented as a set of alternate projection matrices, a different auxiliary matrix is constructed for each of the matrices.

##### Using ranges of vital rates

The user enters a matrix with the ranges of possible values for each vital rate in the projection matrix. When introducing data, only ranges generating positive values and/or transition probabilities smaller than 1 are accepted by the application. This procedure assumes that the vital rates are within an interval of feasible values. Ranges may be given for every vital rate or only for a subset. To extract a projection matrix, vital rates are sampled at random from each interval assuming a uniform distribution of values within the intervals. Implementing random sampling of vital rates is trivial:

(A18) $$A_{i}=\left(A_{m}‐R/2\right)+R\circ P_{i},$$

where ○ denotes the Hadamard product of two matrices and ***A_i_*** is the matrix to be used at run *i*; ***A_m_*** is the average matrix (i.e., the original matrix given as input); ***R*** is a matrix with the ranges of the vital rates (user‐given); and ***P_i_*** is a matrix with randomly selected uniform deviates from a standard uniform distribution, *U*(0, 1). Uniform deviates are at the nonzero entries of matrix ***R***.

## APPENDIX 2. EXAMPLE: Chthamalus montagui IN NORTHERN SPAIN

Data come from unpublished matrices for the barnacle *Chthamalus montagui* Southward in northern Spain. Details for sampling and the construction of the projection matrix are in Suárez & Arrontes (2008). The projection matrix and some demographic parameters are in Table A1. Demographic information and confidence intervals in Table A1 were obtained using Demography_Lab. The projection matrix comes from a postbreeding census. Entries in the first row are fecundities. Reproductive size classes are 2–4. The projection interval was 1 year. The matrix was constructed after the study of the destinations of 203 individuals in class 1 (newly recruited individuals), 127 in class 2, 105 in class 3, and 66 in class 4. *Chthamalus* populations are open populations but, for example, the population was treated as a closed and all 203 recruits were considered to come from reproductives in the area.

Details for simulation to construct Figures 1, 2, 3.

‐ No density dependence was considered.
‐ The threshold for extinction was 1 individual.
‐ Ten thousand replicates were evaluated.
‐ Environmental stochasticity was included by sampling matrix entries from uniform distributions. Ranges for the distribution of each vital rate were: $\left(\begin{array}{cccc} 0.008 & 0.2 & 0.5 & 0.6\\ 0.1 & 0.2 & 0 & 0\\ 0 & 0.1 & 0.2 & 0\\ 0 & 0 & 0.1 & 0.2 \end{array}\right)$.
‐ Demographic stochasticity affected transitions and fecundities. For transitions, multinomial or binomial distributions were sampled. For fecundities, a Poisson distribution was used.
‐ Sampling error in the construction of the projection matrix was implemented by using the bootstrap. At the beginning of each run, a new projection matrix was extracted from the collection of individuals in the original sample of individuals studied.

**TABLE A1:** Matrix used in examples in Figures 1, 2, 3. Matrix is for a population of the intertidal barnacle *Chthamalus montagui* Southward in northern Spain

Parameter	Original value	95 confidence interval	
		Lower values	Upper values
Projection matrix	$\left(\begin{array}{cccc} 0.03 & 0.24 & 0.68 & 1.11\\ 0.24 & 0.52 & 0 & 0\\ 0 & 0.29 & 0.64 & 0\\ 0 & 0 & 0.24 & 0.88 \end{array}\right)$	$\left(\begin{array}{cccc} 0.01 & 0.17 & 0.56 & 0.97\\ 0.18 & 0.43 & 0 & 0\\ 0 & 0.21 & 0.54 & 0\\ 0 & 0 & 0.16 & 0.80 \end{array}\right)$	$\left(\begin{array}{cccc} 0.06 & 0.32 & 0.79 & 1.26\\ 0.30 & 0.61 & 0 & 0\\ 0 & 0.37 & 0.73 & 0\\ 0 & 0 & 0.32 & 0.95 \end{array}\right)$
*λ*	1.03	0.97	1.08
*R_0_*	1.30	—	—
Generation time	10.21	—	—
Damping ratio	1.92	—	—
Stable stage structure	$\left(\begin{array}{c} 0.42\\ 0.20\\ 0.15\\ 0.24 \end{array}\right)$	$\left(\begin{array}{c} 0.39\\ 0.16\\ 0.11\\ 0.19 \end{array}\right)$	$\left(\begin{array}{c} 0.47\\ 0.25\\ 0.20\\ 0.28 \end{array}\right)$
Reproductive value	$\left(\begin{array}{c} 0.05\\ 0.22\\ 0.33\\ 0.40 \end{array}\right)$	$\left(\begin{array}{c} 0.04\\ 0.17\\ 0.29\\ 0.32 \end{array}\right)$	$\left(\begin{array}{c} 0.07\\ 0.26\\ 0.37\\ 0.47 \end{array}\right)$
Fundamental matrix	$\left(\begin{array}{cccc} 1 & 0 & 0 & 0\\ 0.50 & 2.08 & 0 & 0\\ 0.40 & 1.67 & 2.76 & 0\\ 0.79 & 3.26 & 5.39 & 8.20 \end{array}\right)$	—	—
Sensitivity matrix	$\left(\begin{array}{cccc} 0.11 & 0.05 & 0.04 & 0.06\\ 0.43 & 0.21 & 0.15 & 0.25\\ 0.67 & 0.32 & 0.24 & 0.38\\ 0.79 & 0.38 & 0.28 & 0.45 \end{array}\right)$	—	—
Elasticity matrix	$\left(\begin{array}{cccc} 0.01 & 0.01 & 0.02 & 0.07\\ 0.10 & 0.10 & 0 & 0\\ 0 & 0.09 & 0.15 & 0\\ 0 & 0 & 0.07 & 0.39 \end{array}\right)$	—	—

Note

Parameters and confidence intervals were evaluated using Demography_Lab. Note that entries have been rounded to the second decimal place and gross rounding error may occur if demographic parameters are recalculated by the reader. Lower and upper values of vital rates in the projection matrix were independently extracted for each vital rate (same for the entries in the vectors).

## References

[ece37170-bib-0001] Åberg, P. (1992a). A demographic study of two populations of the seaweed, *Ascophyllum nodosum* . Ecology, 73, 1473–14787. 10.2307/1940691

[ece37170-bib-0002] Åberg, P. (1992b). Size‐based demography of the seaweed *Ascophyllum nodosum* in stochastic environments. Ecology, 73, 1488–1501. 10.2307/1940692

[ece37170-bib-0003] Akçakaya, H. R. , Burgman, M. A. , & Ginzburg, L. R. (1999). Applied population ecology. Principles and computer exercises using RAMAS ecolab (2nd ed.). Sinauer.

[ece37170-bib-0004] Angulo, E. , & Villafuerte, R. (2003). Modelling hunting strategies for the conservation of wild rabbit populations. Biological Conservation, 115, 291–301. 10.1016/S0006-3207(03)00148-4

[ece37170-bib-0005] Arrontes, J. (2018). Pop‐Inference: An educational application to evaluate statistical differences among populations. Ecology and Evolution, 8, 5224–5230. 10.1002/ece3.4010 29938044 PMC6010711

[ece37170-bib-0006] Bernstein, R. (2003). Population ecology: An introduction to computer simulations. Wiley.

[ece37170-bib-0007] Botsford, L. W. , White, J. W. , & Hastings, A. (2019). Population dynamics for conservation. Oxford University Press.

[ece37170-bib-0008] Burch, T. K. (2018). Model‐based demography. Essays on integrating data, technique and theory. Springer Open. 10.1007/978-3-319-65433-1 29592788

[ece37170-bib-0009] Caswell, H. (2001). Matrix population models. Construction, analysis, and interpretation (2nd ed.). Sinauer.

[ece37170-bib-0010] Caswell, H. (2019). Sensitivity analysis: Matrix methods in demography and ecology. Springer Open. 10.1007/978-3-030-10534-1

[ece37170-bib-0011] Caswell, H. , Nisbet, R. M. , de Roos, A. M. , & Tuljapurkar, S. (1997). Structured‐population models: Many methods, a few basic concepts. In S. Tuljapurkar , & H. Caswell (Eds.), Structured‐population models in marine, terrestrial, and freshwater systems (pp. 3–17). Chapman and Hall.

[ece37170-bib-0012] Caswell, H. , & Trevisan, M. C. (1994). Sensitivity analysis of periodic matrix models. Ecology, 75, 1299–1303. 10.2307/1937455

[ece37170-bib-0013] Cecco, G. J. , & Gouhier, T. C. (2018). Increased spatial and temporal autocorrelation of temperature under climate change. Scientific Reports, 8, 14850. 10.1038/s41598-018-33217-0 30287852 PMC6172201

[ece37170-bib-0014] Collen, B. , McRae, L. , Deinet, S. , De Palma, A. , Carranza, T. , Cooper, N. , Loh, J. , & Baillie, J. E. M. (2011). Predicting how populations decline to extinction. Philosophical Transactions of the Royal Society, 366, 2577–2586. 10.1098/rstb.2011.0015 PMC3138608 21807738

[ece37170-bib-0015] Crowder, L. B. , Crouse, D. T. , Heppell, S. S. , & Martin, T. H. (1994). Predicting the impact of turtle excluder devices on loggerhead sea turtle populations. Ecological Applications, 4, 437–445. 10.2307/1941948

[ece37170-bib-0016] Darwin, J. H. , & Williams, R. M. (1964). The effect of time of hunting on the size of a rabbit population. New Zealand Journal of Science, 7, 341–352.

[ece37170-bib-0017] Denny, M. (2017). The fallacy of the average: On the ubiquity, utility and continuing novelty of Jensen's inequality. Journal of Experimental Biology, 220, 139–146. 10.1242/jeb.140368 28100801

[ece37170-bib-0018] Doak, D. F. , Gross, K. , & Morris, W. F. (2005). Understanding and predicting the effect of sparse data on demographic analyses. Ecology, 86, 1154–1163. 10.1890/04-0611

[ece37170-bib-0019] Doak, D. F. , Morris, W. F. , Pfister, C. , Kendall, B. E. , & Bruna, E. M. (2005). Correctly estimating how environmental stochasticity influences Fitness and population growth. American Naturalist, 166, E14–E21. 10.1086/430642 15937784

[ece37170-bib-0020] Efron, B. , & Tibshirani, R. J. (1993). An introduction to the bootstrap. Chapman and Hall.

[ece37170-bib-0021] Evans, M. R. , Grimm, V. , Johst, K. , Knuuttila, T. , de Langhe, R. , Lessells, C. M. , Merz, M. , O’Malley, M. A. , Orzack, S. H. , Weisberg, M. , Wilkinson, D. J. , Wolkenhauer, O. , & Benton, T. G. (2013). Do simple models lead to generality in ecology? Trends in Ecology and Evolution, 28, 578–583. 10.1016/j.tree.2013.05.022 23827437

[ece37170-bib-0022] Fieberg, J. , & Ellner, S. P. (2001). Stochastic matrix models for conservation and management: A comparative review of methods. Ecology Letters, 4, 244–266. 10.1046/j.1461-0248.2001.00202.x

[ece37170-bib-0023] Hastings, A. (2005). Unstructured models in ecology: Past, present, and future. In K. Cuddington , & B. E. Beisner (Eds.), Ecological paradigms lost. Routes of theory change (pp. 9–29). Elsevier.

[ece37170-bib-0024] Heino, M. , Ripa, J. , & Kaitala, V. (2000). Extinction risk under coloured environmental noise. Ecography, 23, 177–184. 10.1111/j.1600-0587.2000.tb00273.x

[ece37170-bib-0025] Holling, C. S. (1966). The strategy of building models of complex systems. In K. E. F. Watt (Ed.), Systems analysis in ecology (pp. 195–214). Academic Press.

[ece37170-bib-0026] Johnson, N. L. , & Kotz, S. (1999). Non‐smooth sailing or triangular distributions revisited after some 50 years. The Statistician, 48, 179–187.

[ece37170-bib-0027] Johst, K. , & Wissel, C. (1997). Extinction risk in a temporally correlated fluctuating environment. Theoretical Population Biology, 52, 91–100. 10.1006/tpbi.1997.1322 9356326

[ece37170-bib-0028] Kendall, B. E. , & Fox, G. A. (2002). Variation among individuals and reduced demographic stochasticity. Conservation Biology, 16, 109–116. 10.1046/j.1523-1739.2002.00036.x 35701963

[ece37170-bib-0029] Kendall, B. E. , & Fox, G. A. (2003). Unstructured individual variation and demographic stochasticity. Conservation Biology, 17, 1170–1172. 10.1046/j.1523-1739.2003.02411.x 35701963

[ece37170-bib-0030] Lacy, R. C. , & Pollak, J. P. (2020). Vortex: A stochastic simulation of the extinction process. Version 10.3.8. Chicago Zoological Society.

[ece37170-bib-0031] Lande, R. (2002). Incorporating stochasticity in population viability analysis. In S. Beissinger , & D. R. McCullough (Eds.), Population viability analysis (pp. 18–40). University of Chicago Press.

[ece37170-bib-0032] Lande, R. , Engen, S. , & Saether, B. E. (2003). Stochastic population dynamics in ecology and conservation. Oxford University Press.

[ece37170-bib-0033] Legendre, S. (2020). Projecting populations. In D. L. Murray , & B. K. Sandercock (Eds.), Population ecology in practice (pp. 193–214). Wiley.

[ece37170-bib-0034] Manem, V. S. K. , Kaveh, K. , Kohandel, M. , & Sivaloganathan, S. (2015). Modelling invasion dynamics with spatial random‐fitness due to micro‐environment. PLoS One, 10(10), e0140234. 10.1371/journal.pone.0140234 26509572 PMC4624969

[ece37170-bib-0035] May, R. M. (1973). Stability and complexity in model ecosystems. Princeton University Press. 4723571

[ece37170-bib-0036] McCallum, H. (2000). Population parameters: Estimation for ecological models. Blackwell.

[ece37170-bib-0037] Nakaoka, M. (1996). Dynamics of age‐ and size‐structured populations in fluctuating environments: Applications of stochastic matrix models to natural populations. Researches in Population Ecology, 38, 141–152. 10.1007/BF02515722

[ece37170-bib-0038] Newman, K. B. , & Lindley, S. T. (2006). Accounting for demographic and environmental stochasticity, observation error, and parameter uncertainty in fish population dynamics models. North American Journal of Fisheries Management, 26, 685–701. 10.1577/M05-009.1

[ece37170-bib-0039] Otto, S. P. , & Day, T. (2007). A biologist's guide to mathematical modelling in ecology and evolution. Princeton University Press.

[ece37170-bib-0040] Ranta, E. , Lundberg, P. , & Kaitala, V. (2006). Ecology of populations. Cambridge University Press.

[ece37170-bib-0041] Ripa, J. , & Heino, M. (1999). Linear analysis solves two puzzles in population dynamics: The route to extinction and extinction in coloured environments. Ecology Letters, 2, 219–222. 10.1046/j.1461-0248.1999.00073.x

[ece37170-bib-0042] Ripa, J. , & Lundberg, P. (1996). Noise colour and the risk of population extinctions. Proceedings of the Royal Society London B, 263, 1751–1753. 10.1098/rspb.1996.0256

[ece37170-bib-0043] Ruel, J. J. , & Ayres, M. P. (1999). Jensen's inequality predicts effects of environmental variation. Trends in Ecology and Evolution, 14, 361–366. 10.1016/s0169-5347(99)01664-x 10441312

[ece37170-bib-0044] Ruokolainen, L. , Lindén, A. , Kaitala, V. , & Fowler, M. S. (2009). Ecological and evolutionary dynamics under coloured environmental variation. Trends in Ecology and Evolution, 24, 555–563. 10.1016/j.tree.2009.04.009 19699550

[ece37170-bib-0045] Schwager, M. , Johst, K. , & Jeltsch, F. (2006). Does red noise increase or decrease extinction risk? Single extreme events versus series of unfavorable conditions. American Naturalist, 167, 879–888. 10.1086/503609 16615033

[ece37170-bib-0046] Shaffer, M. (1981). Minimum population sizes for species conservation. BioScience, 31, 131–134. 10.2307/1308256

[ece37170-bib-0047] Shaffer, M. (1987). Minimum viable populations: Coping with uncertainty. In M. E. Soulé (Ed.), Viable populations for conservation (pp. 69–86). Cambridge University Press.

[ece37170-bib-0048] Smith, G. C. , & Trout, R. C. (1994). Using Leslie matrices to determine wild rabbit population growth and the potential for control. Journal of Applied Ecology, 31, 223–230. 10.2307/2404538

[ece37170-bib-0049] Suárez, R. , & Arrontes, J. (2008). Population dynamics of the barnacle *Chthamalus montagui* at two spatial and temporal scales in northern Spain. MarineBiology, 155, 363‐374. 10.1007/s00227-008-1032

[ece37170-bib-0050] Thompson, W. L. (2004). Sampling rare or elusive species. Concepts, designs, and techniques for estimating population parameters. Islands Press.

[ece37170-bib-0051] Tuljapurkar, S. (1990). Population dynamics in variable environments. Springer. 10.1016/0040-5809(85)90019-x 4060082

[ece37170-bib-0052] Tuljapurkar, S. , & Haridas, C. V. (2006). Temporal autocorrelation and stochastic population growth. Ecology Letters, 9, 327–337. 10.1111/j.1461-0248.2006.00881.x 16958899

[ece37170-bib-0053] Turchin, P. (2003). Complex population dynamics. A theoretical/empirical synthesis. Princeton University Press.

[ece37170-bib-0054] Vasseur, D. A. , DeLong, J. P. , Gilbert, B. , Greig, H. S. , Harley, C. D. G. , McCann, K. S. , Savage, V. , Tunney, T. D. , & O’Connor, M. I. (2014). Increased temperature variation poses a greater risk to species than climate warming. Proceedings of the Royal Society B, 282, 20132612. 10.1098/rspb.2013.2612 PMC3924069 24478296

[ece37170-bib-0055] Vavrek, M. C. , McGraw, J. B. , & Yang, H. S. (1997). Within‐population variation in demography of *Taraxacum officinale*: Season‐ and size‐dependent survival, growth and reproduction. Journal of Ecology, 85, 277–287. 10.2307/2960501

[ece37170-bib-0056] Young, T. P. (1994). Natural die‐offs of large mammals: Implications for conservation. Conservation Biology, 8, 410–418. 10.1046/j.1523-1739.1994.08020410.x

